# Carbon-Doped Co_2_MnSi Heusler Alloy Microwires with Improved Thermal Characteristics of Magnetization for Multifunctional Applications

**DOI:** 10.3390/ma16155333

**Published:** 2023-07-29

**Authors:** Mohamed Salaheldeen, Asma Wederni, Mihail Ipatov, Valentina Zhukova, Arcady Zhukov

**Affiliations:** 1 Department of Polymers and Advanced Materials, Faculty of Chemistry, University of the Basque Country (UPV/EHU), 20018 San Sebastián, Spain; asma.wederni@ehu.eus (A.W.); mihail.ipatov@ehu.eus (M.I.); valentina.zhukova@ehu.eus (V.Z.); 2 Department of Applied Physics I, EIG, University of the Basque Country (UPV/EHU), 20018 San Sebastián, Spain; 3 Physics Department, Faculty of Science, Sohag University, Sohag 82524, Egypt; 4 EHU Quantum Center, University of the Basque Country (UPV/EHU), 20018 San Sebastián, Spain; 5 IKERBASQUE—Basque Foundation for Science, 48011 Bilbao, Spain

**Keywords:** Heusler alloys, glass-coated microwires, thermal stability, magnetic sensing, HR-TEM

## Abstract

In the current work, we illustrate the effect of adding a small amount of carbon to very common Co_2_MnSi Heusler alloy-based glass-coated microwires. A significant change in the magnetic and structure structural properties was observed for the new alloy Co_2_MnSiC compared to the Co_2_MnSi alloy. Magneto-structural investigations were performed to clarify the main physical parameters, i.e., structural and magnetic parameters, at a wide range of measuring temperatures. The XRD analysis illustrated the well-defined crystalline structure with average grain size (D_g_ = 29.16 nm) and a uniform cubic structure with A2 type compared to the mixed L2_1_ and B2 cubic structures for Co_2_MnSi-based glass-coated microwires. The magnetic behavior was investigated at a temperature range of 5 to 300 K and under an applied external magnetic field (50 Oe to 20 kOe). The thermomagnetic behavior of Co_2_MnSiC glass-coated microwires shows a perfectly stable behavior for a temperature range from 300 K to 5 K. By studying the field cooling (FC) and field heating (FH) magnetization curves at a wide range of applied external magnetic fields, we detected a critical magnetic field (H = 1 kOe) where FC and FH curves have a stable magnetic behavior for the Co_2_MnSiC sample; such stability was not found in the Co_2_MnSi sample. We proposed a phenomenal expression to estimate the magnetization thermal stability, ΔM (%), of FC and FH magnetization curves, and the maximum value was detected at the critical magnetic field where ΔM (%) ≈ 98%. The promising magnetic stability of Co_2_MnSiC glass-coated microwires with temperature is due to the changing of the microstructure induced by the addition of carbon, as the A2-type structure shows a unique stability in response to variation in the temperature and the external magnetic field. In addition, a unique internal mechanical stress was induced during the fabrication process and played a role in controlling magnetic behavior with the temperature and external magnetic field. The obtained results make Co_2_MnSiC a promising candidate for magnetic sensing devices based on Heusler glass-coated microwires.

## 1. Introduction

Nano- and microstructure magnetic materials offer special physical characteristics that make them suitable for a variety of industrial applications, including information technology, energy, and healthcare. They are utilized in the creation of computer memory, MRI machines, spintronic devices, magnetic refrigeration, hard disk drives, magnetic sensors, and renewable energy sources [1,2,3,4]. Their special qualities make them a fantastic substitute for traditional materials and have the ability to completely transform a variety of sectors by making them more effective, economical, and environmentally friendly.

Magnetic Heusler alloys are a class of materials that have gained significant attention due to their unique magnetic properties [5,6]. These alloys are composed of transition metals such as cobalt, iron, and nickel and are known for their half-metallic behavior, meaning that they have a high electrical conductivity in one spin channel and a low electrical conductivity in the other [7]. In addition, Heusler alloys can have a high magnetization and a high Curie temperature, making them resistant to demagnetization at high temperatures [8]. These properties make Heusler alloys promising candidates for use in a wide range of applications, including magnetic storage media, sensors, and energy-efficient motors [5,9,10]. However, further research is needed to fully understand these materials’ behavior and to optimize their properties for practical use.

Co_2_Mn-based Heusler alloys are a type of intermetallic compound that is composed of cobalt, manganese, and a small amount of a third element, such as aluminum or silicon. These alloys are known for their interesting magnetic and electronic properties, which make them of interest for a variety of applications, including in spintronic devices, sensors, and energy-efficient motors [9,10,11].

One of the most notable properties of Co_2_Mn-based Heusler alloys (especially Co_2_MnSi), is that they can exhibit half-metallic behavior, meaning that they have a high density of states at the Fermi level for one spin channel, but not the other [9,12,13]. These alloys are widely recognized for their large bandgap for minority spins (0.5 to 0.8 eV), high Curie temperature (∼985 K), high tunnel magnetoresistance, large magnetoresistance ratios, and perpendicular magnetic anisotropy [11,12,13,14]. Both experimental and theoretical investigations conducted on Co_2_MnSi in the last two decades have focused on the analysis of structural and magnetic properties and their relation to spin polarization [12,13,14,15]. The highest value of spin polarization for bulk Co_2_MnSi, ∼93%, was measured at room temperature by ultraviolet-photoemission spectroscopy [15]. These properties are useful for a variety of applications, including cutting tools and wear-resistant coatings. Co_2_Mn-based Heusler alloys can be produced through various methods, including powder metallurgy, spark plasma sintering, and hot isostatic pressing [15]. Doping the alloy with concordant atoms is one of the suitable methods for tuning the bandgap value of Heusler alloys [16]. Therefore, for the current study, we wanted to investigate the effect of adding carbon to the Co_2_MnSi alloy on the magneto-structural properties. Carbon addition, used to improve phase stability and coercivity, leads to the deformation of the unit cell and can affect the Mn-Mn coupling [17,18]. Thus, we present a primary investigation of magneto-structural properties of Co_2_MnSiC-based glass-coated microwires. The choice of the glass-coated microwire physical form is due to the interesting magneto-structural behavior of Heusler-based glass-coated microwire [19,20,21,22,23,24,25,26].

Co_2_MnSiC glass-coated microwires, studied in the current paper, are prepared by using the Taylor–Ulitovsky method developed in the 1960s [27]. The Taylor–Ulitovsky method involves the rapid quenching processes used to prepare Heusler alloy glass-coated microwires [19,20,21,22,23]. Initially, this technique was developed for the preparation of non-magnetic glass-coated microwires [27]. However, since the 1970s, almost the same preparation method has been employed for the preparation of amorphous magnetic microwires [28,29,30,31]. Recently, the preparation of glass-coated microwires with metallic nucleus diameters ranging from 0.5 to 100 µm using this technology was reported by several authors [29,30,31,32,33,34,35,36,37,38]. The main benefit of this low-cost preparation method is that it allows the rapid (up to a few hundred meters per minute) production of thin and long (up to a few kilometers) microwires with a wide diameter range. This method is also suitable for the preparation of glass-coated microwires with improved mechanical properties [30]. The glass coating on the microwires can provide additional benefits, such as improved insulation, protection against environmental factors, and improved mechanical properties of fragile crystalline alloys [30]. Furthermore, biological applications would benefit from the availability of a biocompatible, thin, flexible, insulating, and highly transparent glass coating [31]. Additionally, the Taylor–Ulitovsky fabrication technique provides the unique possibility of miniaturizing the Heusler alloys: microwires that are long and only a few micrometers in diameter can be prepared directly from an ingot. The Heusler alloy miniaturization is, in fact, one of the issues from the viewpoint of device and sensor development [39]. Thus, the heat exchange rate can be substantially improved by increasing the surface-to-volume ratio by using thin ribbons, films, or wires. Accordingly, Co_2_MnSiC-based Heusler microwires are a promising smart material for a wide range of technological applications. As far as we are aware, the production and structural, mechanical, and magnetic characterization of Co_2_MnSiC-based Heusler glass-covered microwires have not been substantially examined. The structural and magnetic properties of Co_2_MnSiC microwires will thus be the primary focus of the current work to demonstrate their potential applications in cutting-edge spintronics.

In the current study, we want to highlight for the first time the magneto-structural properties of Co_2_MnSiC and the effect of the external magnetic field and the temperature on its magnetic behavior. Unique magnetization thermal stability has been reported for a wide range of temperatures (5–300 K) and magnetic fields. In addition, we detected a critical magnetic field where the magnetization curves show perfect thermal stability. The unique magnetic properties together with the other well-known physical properties of Co_2_MnSiC-based glass-coated microwires make them a promising candidate for many interesting multifunctional applications.

## 2. Materials and Methods

For preparing the Co_2_MnSiC alloy, we followed the same procedures reported in [21,35], but with the addition of carbon with a proper percentage. High-purity cobalt (99.99%) (50 at.%), manganese (99.9%) (24.6 at.%), silicon (99.99%) (25 at.%), and carbon (99.9%) (0.4 at.%) were weighed and placed in a ceramic crucible. Then, we used an arc furnace to melt the mixture of the alloy under a vacuum to prevent oxidation. The melting process was repeated 5 times to obtain a homogeneous Co_2_MnSiC alloy. Once the Co_2_MnSiC alloy was ready, the ingot moved to the next step where we could fabricate Co_2_MnSiC microwires covered by insulating (Duran) glass coating using the Taylor–Ulitovsky method. The Taylor–Ulitovsky method has several advantages over other methods for preparing glass-coated microwires. One advantage is that it allows for the preparation of microwires with a very thin glass coating, typically a few micrometers in thickness. This thin coating allows for the preservation of the electrical and magnetic properties of the microwire metallic nucleus, making the resulting microwires useful for a variety of applications. The fabrication process is described in detail in several previous works [19,20,21,35,38]. The diameter of the metallic nuclei, d, was then determined by controlling the speed of wire drowning, molten alloy temperature, and receiving bobbin rotation speed. To complete the quick melt-quenching process, the produced microwire was passed through a coolant stream [21,35,36]. Through scanning electron microscopy (SEM), we determined that Co_2_MnSiC glass-coated microwires have a metallic nucleus diameter, d, of 13.89 µm and total diameter D_total_ = 17.31 µm with an aspect ratio *ρ* = d/D_total_ = 0.80. This manufacturing method is particularly beneficial for alloys containing Mn due to fast alloy solidification, allowing it to protect against oxidation by the insulating glass coating [21,40]. Therefore, this procedure proves suitable for the production of such materials, while achieving desired results in terms of quality control.

For investigating the magnetic properties of the Co_2_MnSiC-based glass-coated microwires, we used a Physical Property Measurement System (PPMS) (Quantum Design Inc., San Diego, CA, USA). We measured the magnetization curves for magnetic field, H, parallel to the wire axis, where the easy magnetization axis is expected due to the shape of magnetic anisotropy. The measurements were performed at a wide range of temperatures (5–300 K) and magnetic field strengths (50 Oe–20 kOe). In addition, we studied the magnetic behavior under zero-field cooling, heating, and cooling fields to assess the possible magnetic phase transition or irreversibility behavior. The morphological and chemical composition and microstructure were studied by using energy-dispersive X-ray spectroscopy (EDX) and X-ray diffraction (XRD) (BRUKER D8 Advance, Bruker AXS GmbH, Karlsruhe, Germany). Cu K_α_ (λ = 1.54 Å) radiation was used in all the patterns. For microstructure investigation, we used high-resolution transmission electron microscopy (HR-TEM) (JEOL JEM2100, JEOL, Tokyo, Japan).

## 3. Results

### 3.1. Structural Properties

Table 1 shows the results of an EDX/SEM examination conducted to determine the chemical composition of Co_2_MnSiC glass-coated microwires and compares them with the results for the Co_2_MnSi sample. The composition of the metallic nucleus evaluated by EDX/SEM is somewhat different from the stoichiometric one (Co_2_MnSiC). This slight variation was caused by the preparation procedure’s characteristics, which comprised alloy melting and casting. We evaluated the actual composition of ten different places to determine the extent of the variation. The atomic average composition of Co_50.4_Mn_23.6_Si_25.6_C_0.4_ for Co_2_MnSiC was validated for all sites. An elevated Si content is attributed to the interfacial layer between the glass coating and the metallic nucleus [40,41]. The Co, Mn, and Si at.% values are almost similar for Co_2_MnSiC and Co_2_MnSi alloys (see Table 1).

**Figure 1:**
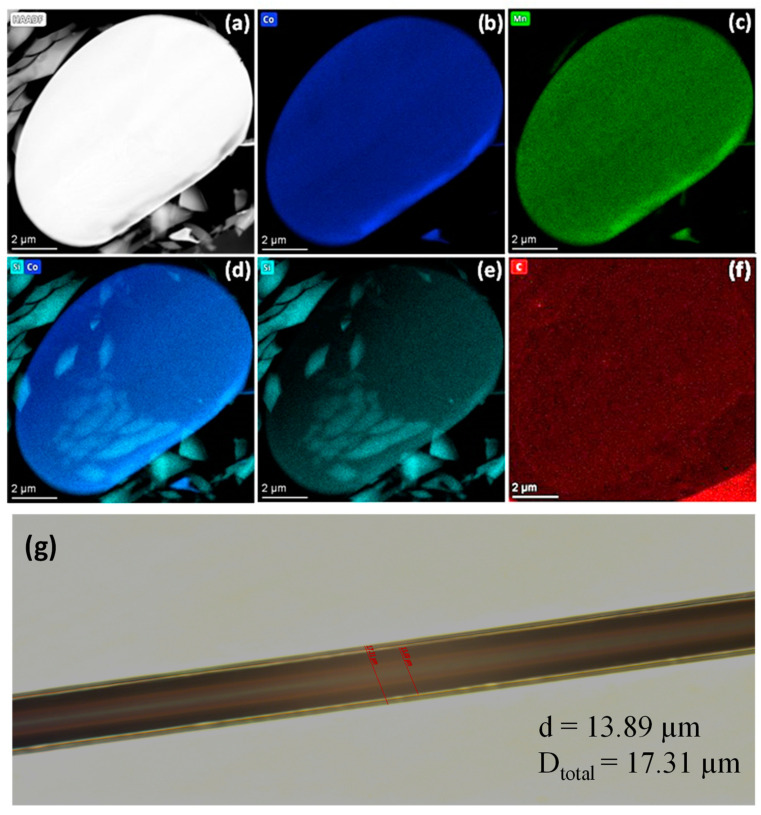
TEM image (**a**–**f**) with energy-dispersive X-ray (EDX) mapping for single Co_2_MnSiC glass-coated microwires for Co, Mn, Si, and C. (**g**) The optical microscope image of the synthesized Co_2_MnSiC microwires.

To confirm the chemical structure composition and distribution in Co_2_MnSiC glass-coated microwires, we performed the mapping of the elements by using the high-resolution transmission electron microscopy (TEM) supported by EDX. Figure 1 illustrates the homogeneous distribution of Co, Mn, Si, and C elements in a single Co_2_MnSiC glass-coated microwire. The image cross-section does not show a perfect circular shape; the distortion is due to the not exactly perpendicular cutting process, which results in an oval image shape. In addition, at the edge of the image, a more contracted color is shown due to either distortion or the interfacial layer, but in the rest of the microwire, a perfect homogeneous distribution is obtained. The fine details that appear in Figure 1d,e come from small pieces of glass coating, as evidenced by the increase in the Si percentage content. As seen in Figure 1f, a homogeneous carbon distribution in the microwires is generally observed. However, an additional amount of carbon appears outside the cross-section. Similarly to that observed previously, such elevated carbon content outside the metallic nucleus must be attributed to the defects originated by the interface layer as well as by the sample preparation for the TEM evaluation [41]. 

**Figure 2:**
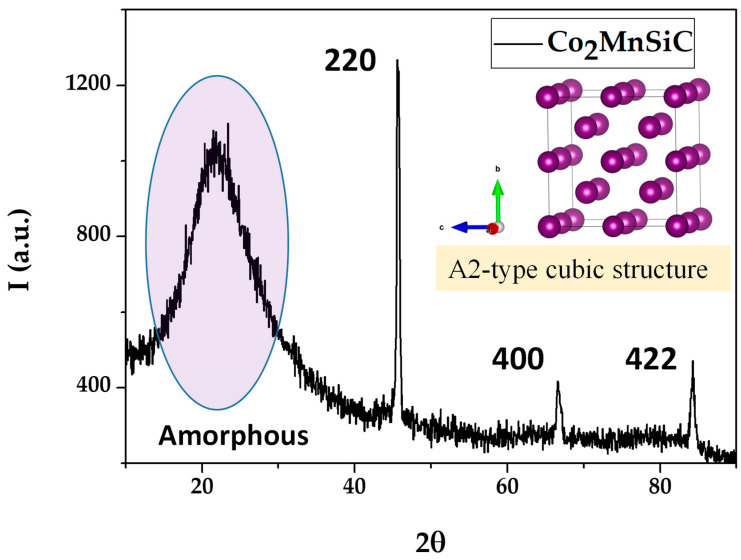
X-ray diffraction profile of Co_2_MnSiC glass-coated microwires measured at room temperature.

Figure 2 illustrates the X-ray diffraction (XRD) patterns of the Co_2_MnSiC glass-coated microwires measured at room temperature (RT). All Miller indices are labeled on the patterns. As illustrated in Figure 1, there is a wide halo at 2θ ≈ 22°, commonly attributed to the presence of an amorphous glass-coating layer, also observed in our previous works [19,21]. The presence of (220), (400), and (422) peaks in the XRD pattern must be attributed to the cubic structure [42]. Accordingly, the presence of the austenite phase is expected at room temperature in the studied Co_2_MnSiC samples.

As a result, the entire diffraction pattern has been successfully identified by the existence of the cubic austenite structure. We should state that the lack of (111) and (200) superlattice diffraction peaks confirms the presence of an A2-type cubic structure [42,43]. Indeed, no secondary phase was detected in all the XRD patterns. To evaluate the grain size, D_g_, related to each peak, we used the Debye Scherrer formula [44,45]:

D_g_ = K λ/β cos2θ (1)

where K is a dimensionless form factor with a value of roughly 0.94 (which might vary depending on the actual shape of the crystallite), and the experimental XRD wavelength (Cu-K (alpha) = 1.54) and β present the whole width at half maximum of the XRD peaks. Table 2 summarizes the differences in the microstructure between the Co_2_MnSiC and Co_2_MnSi glass-coated microwires, where a notable reduction in D_g_ and the lattice parameter is observed.

The average D_g_ is about 29.2 nm, which is lower than that we reported for Co_2_MnSi-based glass-coated microwires (D_g_ = 46 nm). The reduced D_g_ value can be related to several factors, such as doping by a small amount of carbon or a higher quenching rate due to thinner glass-coating thickness (0.4 µm for Co_2_MnSiC microwire versus 5 µm for Co_2_MnSi microwire). As discussed elsewhere, the average grain size substantially affects the magnetic properties of nanocrystalline materials [45]. Accordingly, such a reduced D_g_ value can substantially affect the magnetic properties of Co_2_MnSiC glass-coated microwires, as will be illustrated in the magnetic characterization part.

### 3.2. Microstructural Investigation

**Figure 3:**
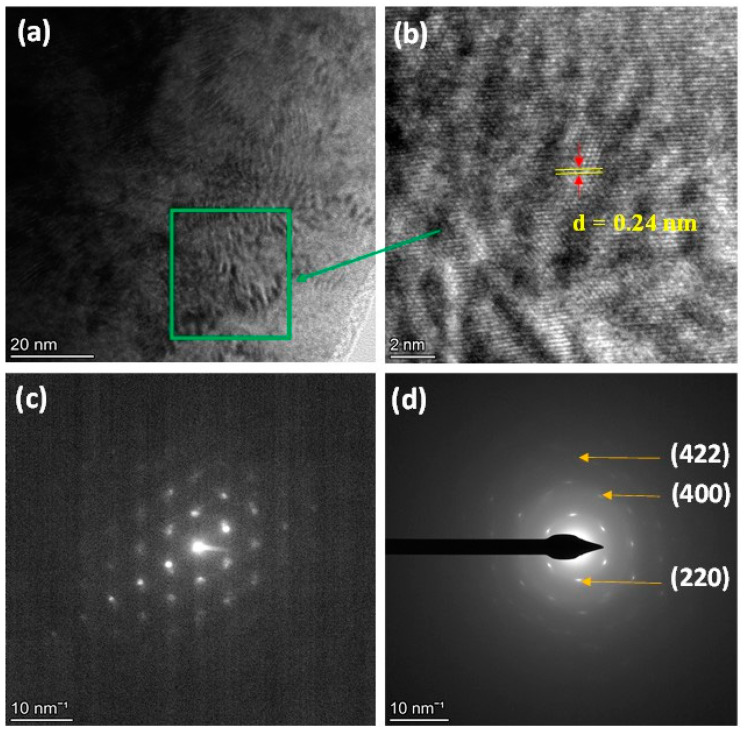
(**a**,**b**) HR-TEM image of Co_2_MnSiC glass-coated microwires for green rectangular region; (**c**,**d**) FFT and SAED pattern acquired from rectangle region.

In this section, we only concentrate on the microstructure investigation of Co_2_MnSiC to confirm its initial properties and agree with the XRD finding. Figure 3 shows the selected area electron diffraction image of single Co_2_MnSiC glass-coated microwires obtained by HR-TEM. As illustrated in Figure 3a,b, there is an evident crystalline phase with an interplanar spacing of 0.24 nm. The fast Fourier transform (FFT) and SAED pattern confirm the cubic structure (see Figure 3c,d). The first three rings can be indexed with the (hkl) values (220), (400), and (422), which are consistent with the XRD results (see Figure 2). The clearly visible lattice bright points confirm the high crystallinity of the Co_2_MnSiC. The interplanar spacing of 0.24 nm is equivalent to the (220) plane of the cubic Heusler phase of Co_2_MnSi [46]. The main difference between the microstructures of Co_2_MnSi and Co_2_MnSiC is a fully disordered A2 cubic structure, as compared to the L2_1_ (ordered) and B2 (disordered) cubic structure observed in Co_2_MnSi microwires [47,48,49,50,51,52]. Such difference can be related to either the carbon doping or different fabrication conditions mainly associated with the thinner glass coating for the Co_2_MnSiC microwire.

### 3.3. Magnetic Properties

**Figure 4:**
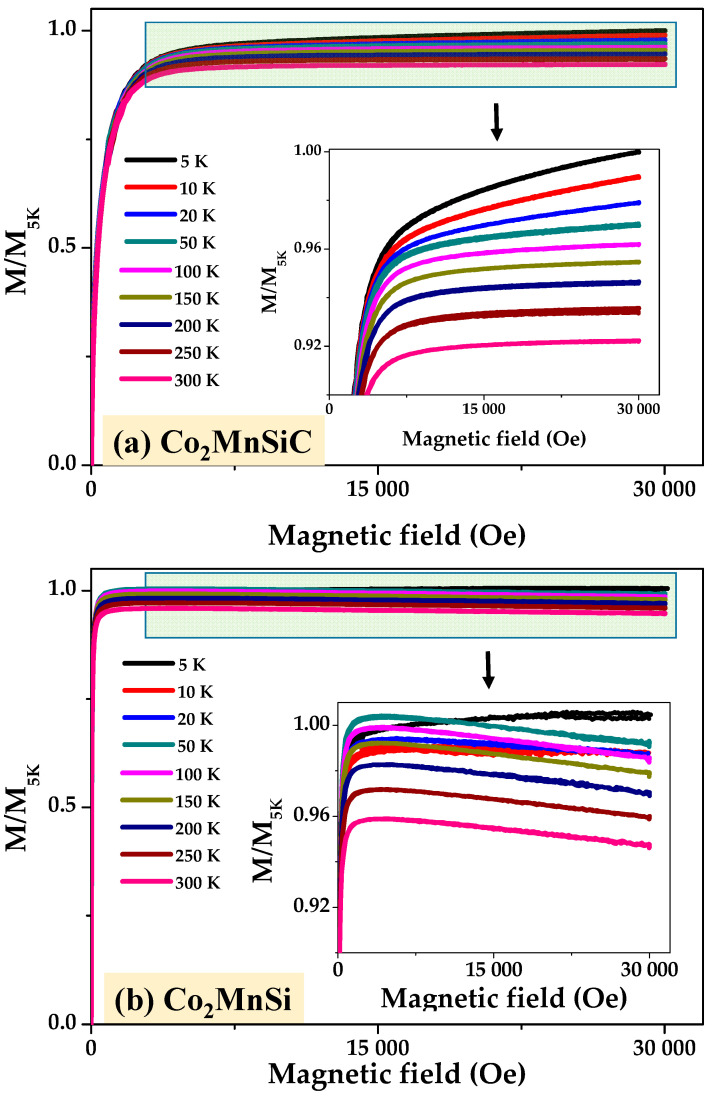
Magnetization (M/M_5K_) vs. magnetic field curves of as-prepared Co_2_MnSiC (**a**,**b**) and Co_2_MnSi glass-coated microwires measured at the temperature range of 5 to 300 K. Inset illustrates the high magnification of magnetic curves with temperature, where the paramagnetic effect starts to appear at T = 50 K for Co_2_MnSiC and the antiferromagnetic effect for Co_2_MnSi-glass coated microwires occurs at T < 50 K.

This section deals with the magnetic behavior studied between 300 K and 5 K. As described in the experimental section, we employed the PPMS to explore the magnetic properties of Co_2_MnSiC and Co_2_MnSi glass-coated microwires over wide temperature, T, and magnetic field, H, ranges. Figure 4 depicts the M/M_5K_ (H) curves, measured at various temperatures. The M/M_5K_ (H) loops exhibit ideal saturated curves between 300 and 50 K; however, at T 50 K, a noticeable deviation from the saturation begins to occur. Such deviation increases with the decrease in T (see the inset of Figure 4a). The peculiarity of the Co_2_MnSiC microwires with respect to at Co_2_MnSi is the presence of a fully disordered microstructure with A2 type, as described in Figure 2 and Figure 3. This A2-type microstructure breaks the antiferromagnetic order of Mn-Mn and enhances the paramagnetic effect at temperatures below 50 K. For samples without carbon, strong antiferromagnetic Mn-Mn coupling has been detected (see Figure 4b); for more, details see Ref. [21].

**Figure 5:**
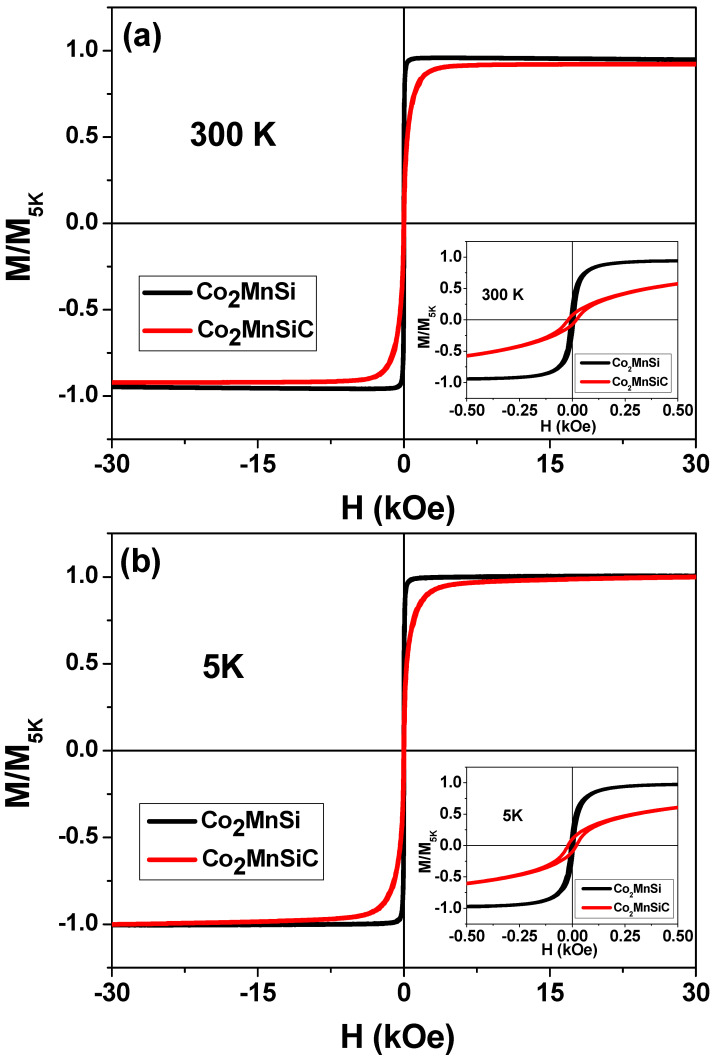
(**a**,**b**) M-H hysteresis loops, measured in an applied magnetic field (±30 kOe) parallel to the axis of the microwires at different temperatures from 5 K to 300 K for as-prepared Co_2_MnSi and Co_2_MnSiC glass-coated microwires, respectively. The insets show the magnetic field and M/M_5K_ at a low magnification scale.

The complete M/M_5K_ (H) curves for Co_2_MnSi and Co_2_MnSiC glass-coated microwires are shown in Figure 5. Such M/M_5K_ (H) loops, measured at the magnetic field of ±30 kOe, almost perfectly match at temperatures 300–5K. Small differences were observed only at the saturation part of the M-H loops, as discussed in the previous paragraph. As illustrated in Figure 5a,b, the Co_2_MnSiC sample shows a higher coercivity and lower normalized remanent compared to the Co_2_MnSi sample at low and high temperatures. These variations are due to the changing of the microstructure, which affects the magnetic microstructure of the sample and its response to variations in the temperature and the magnetic field. 

The main magnetic parameters, such as coercivity, Hc, and magnetic remanence, M_r_, extracted from low-field M/M_5K_ (H) loops measured at different temperatures are shown in Table 3. From M/M_5K_ (H) loops, we can deduce low Hc values showing an average Hc ≈ 19.4 Oe for the Co_2_MnSiC sample and the average of Hc ≈ 6.9 Oe for the Co_2_MnSi sample at all ranges of measuring temperatures, illustrating the soft magnetic properties of the studied microwire. The temperature dependence of Hc and M_r_ show unique stability with temperature (see Table 3). The in-plane coercivity of Co_2_MnSiC glass-coated microwires shows a rather stable Hc value, where the difference between the lowest and the highest value of Hc, i.e., ΔHc, is around 0.3 Oe (compared to 4 Oe for the sample without carbon). In addition, the difference between the normalized M_r_ (max) and normalized M_r_ (mini), ΔM_r_, is about 0.03, as shown in Table 3. The observed unusual high-temperature stability of Hc and M_r_ makes this new alloy, i.e., Co_2_MnSiC glass-coated microwires, promising for application in magnetic sensing. For Co_2_MnSi-based glass-coated microwires, i.e., without carbon doping, the Hc and M_r_ temperature dependencies also show a quite stable behavior, but ΔHc is around 4 Oe and ΔM_r_ = 0.05. Therefore, the studied Co_2_MnSiC microwires present better thermal stability of Hc, which can be attributed to the carbon doping of the Co_2_MnSi glass-coated microwires or the higher quenching rate associated with the thinner glass coating. Accordingly, the energy loss of the ferromagnetic materials becomes stable for a temperature range of 300 to 5 K, which is very important for magnetic storage media, sensors, and energy-efficient motor devices. 

**Figure 6:**
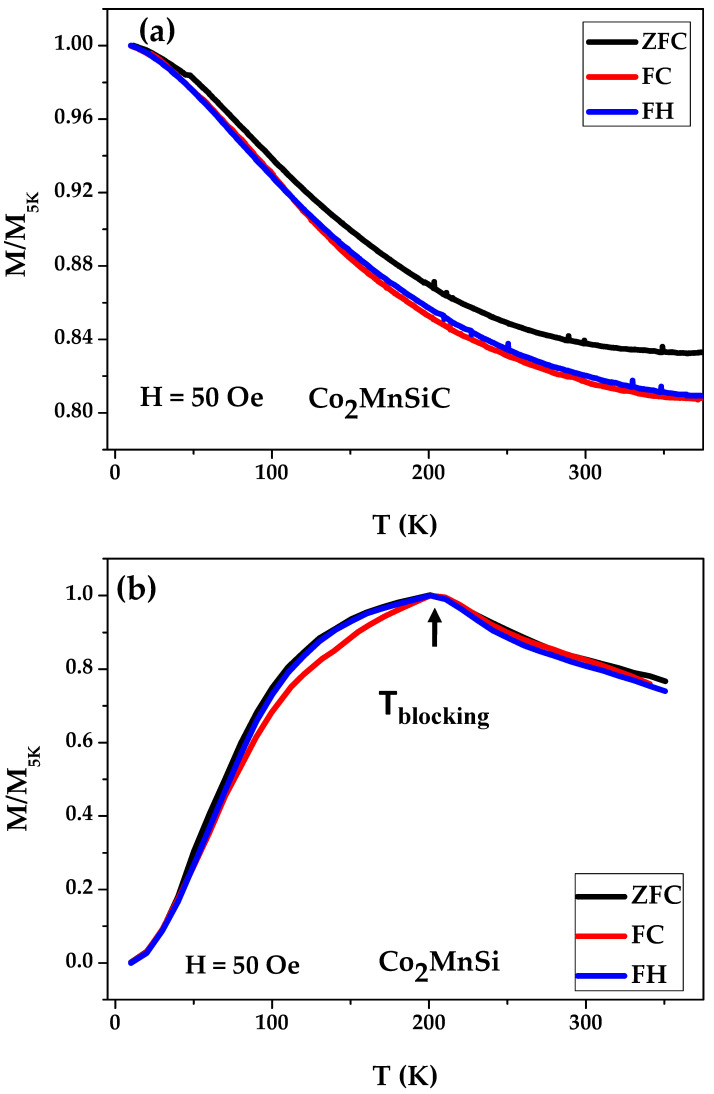
Zero-field cooling (ZFC), field cooling (FC), and field heating (FH) of as-prepared Co_2_MnSiC (**a**,**b**) and Co_2_MnSi glass-coated microwires.

**Figure 7:**
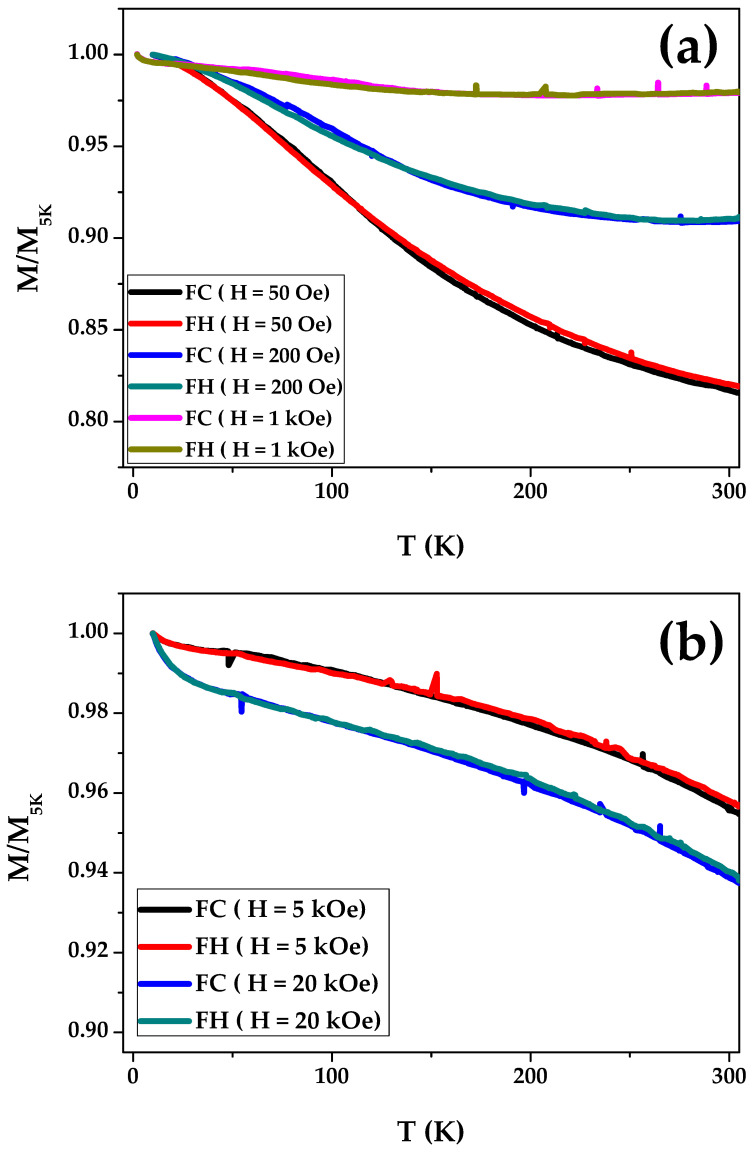
Temperature dependence of magnetization (M/M_5K_), field cooling (FC), and field heating (FH) measured for as-prepared Co_2_MnSiC glass-coated microwires with applied external magnetic field: (**a**) H = 50 Oe, 200 Oe, and 1 kOe; (**b**) H = 5 kOe and 20 kOe.

It is critical to analyze the magnetic behavior with temperature in detail in order to examine thermal stability, which is a critical physical quality in determining the potential for spintronic applications. Furthermore, the temperature dependence of magnetization can provide important information on magnetic phase transformation. The magnetization dependence versus temperature (M vs. T), i.e., zero-field cooling (ZFC) and field cooling (FC), over a wide range of magnetic field strengths (H = 50 Oe to 20 kOe) and temperatures (5 to 300 K) is shown in Figure 6 and Figure 7. The as-prepared Co_2_MnSiC and Co_2_MnSi glass-coated microwires were cooled down from 300 K to 5 K under an applied low magnetic field (H = 50 Oe) in the field cooling protocol, causing the random magnetic moment vectors to freeze parallel to the applied field at low temperatures. Figure 6 shows the ZFC, FC, and FH measured under a low magnetic field. For the Co_2_MnSiC sample, all magnetization curves show perfect ferromagnetic behavior without any magnetic phase transition, where the M/M_5K_ ratio has a monotonic increase as the temperature decreases from 300 K to 5 K. The differences between the M/M_5K (300K)_ and M/M_5K (5K)_ are (ΔM/M_5K_) ZFC = 0.16, (ΔM/M_5K_) FC = 0.19, and (ΔM/M_5K_) FH = 0.18. Such small differences in the ΔM/M_5K_ between the ZFC, FC, and FH magnetization curves must be related to the change in the internal stresses originated mainly by the glass coating under the change in the magnetic field and the temperature. The origin of internal stresses in glass-coated microwires is discussed in detail elsewhere [53,54,55]. The main source of the internal stresses is the difference in the thermal expansion coefficients of metallic alloy solidifying inside the glass coating [53,54,55]. The magnitude of such internal stresses can reach 1 GPa [53,54,55]. The other sources, such as the quenching stresses related to the rapid quenching of the metallic alloy and the drawing stresses, are usually an order of magnitude lower [53,54,55]. Meanwhile, for Co_2_MnSi glass-coated microwires, large irreversibility with a blocking temperature T = 150 K has been observed, as shown in Figure 6b. This irreversibility is stable under the application of an external magnetic field from 50 Oe to 20 kOe. This behavior illustrates the strong influence of carbon in changing the magnetic properties and occurs at different magnetic fields and temperatures.

Figure 7 depicts FC and FH applied at various magnetic fields ranging from 50 Oe to 20 kOe. All FC and FH magnetization curves exhibit ferromagnetic behavior over the entire temperature range. Magnetization curves, measured at low magnetic fields, such as 50 Oe and 200 Oe, present strong modifications with temperature. The slope on M/M_5K_ (T) vanished when the applied external magnetic field was increased up to 1 kOe, and the FC and FH curves became almost straight (see Figure 7a). Figure 7b shows how the FC and FH magnetization curves behave when an external magnetic field is applied. The M/M_5K_ (T) dependencies measured at different H values illustrate the sensitivity of Co_2_MnSiC glass-coated microwires to the temperature and the external magnetic field.

The studied microwires have a nanocrystalline structure and relatively low *H_c_* (about 5–10 Oe). However, such *H_c_* values are about 2 orders of magnitude higher than those for amorphous microwires [56]. In spite of relatively low coercivity, a substantial effect of the applied field on M/M5K(T) dependencies is observed (see Figure 7). Such a substantial magnetic field dependence of the M/M5K(T) was previously reported for other Heusler alloy microwires (NiMnGa) and was attributed to a nonuniform magnetic character of the microwires produced by the Taylor–Ulitovsky method and explained by the atomic disorder and magnetic clustering [56,57].

From the FC and FH magnetization curves of Co_2_MnSiC glass-coated microwires measured at different magnetic fields, we can estimate the magnetization thermal stability (ΔM) of each of the FC and FH magnetization curves of Co_2_MnSiC glass-coated microwires. We proposed a phenomenal formula of ΔM which depends on the difference between the maximum value of the magnetization and the minimum value of the magnetization at a specific range of temperature. As all FC and FH curves show ferromagnetic behavior, the maximum value of M/M5K measured at 5 K, and the lowest value of M/M_5K_ measured at T = 300 K, we can estimate the ΔM (%) for ΔT (the range of measuring temperature, i.e., 5–300 K) by using the following formula:

ΔM (%) = (M/M_5K_ − ((M/M_5K_) _(T=5K)_ − ((M/M_5K_) _(T=300K)_)) × 100

i.e., ΔM (%) = (1 − ΔM/M_5K_) × 100

All calculated values are summarized in Table 4.

As illustrated in Table 4, the minimum thermal magnetization stability is detected for FC and FH magnetization curves at H = 50 Oe, at which it is over 80%. The highest ΔM is observed at H = 1 kOe, at which ΔM is near 98%; i.e., the change in the M/M_5K_ magnetization ratio with temperature is only 2%, which means very high magnetization thermal stability. In addition, the average magnetization thermal stability for all magnetic field ranges is about 92%. Such behavior was not observed in Co_2_MnSi glass-coated microwires, as the FC and FH magnetization curves of Co_2_MnSi microwires show a large irreversibility magnetic behavior at low temperatures. Thus, ΔM for Co_2_MnSi glass-coated microwires has a low-temperature stability as compared to the Co_2_MnSiC glass-coated microwires. Therefore, the studied Co_2_MnSiC glass-coated microwires are a suitable candidate for micro-motors and generator devices based on glass-coated microwires. In addition, glass-coated microwires can be widely applied in mobile sensing and green energy applications.

## 4. Conclusions

In summary, we studied the magneto-structural properties of novel Co_2_Mn Heusler alloy-based glass-coated microwires (Co_2_MnSiC) prepared by using the Taylor–Ulitovsky method. The structure analysis proves the formation of a nanocrystalline structure with an A2-type cubic structure due to the lack of (111) and (200) superlattice peaks. The magnetic measurements reveal the unique thermal stability over a wide range of temperatures, 300 –5K, where the H_c_ and M_r_ show an almost stable tendency with decreasing temperature. ZFC, FC, and FH magnetization curves show a regular ferromagnetic behavior when the temperature is decreased from 400 K to 5 K under the applied external magnetic field (H = 50 Oe and 200 Oe). Under the magnetic field of 1 kOe, FC and FH magnetization curves show the lowest change with temperature. The unique thermal stability of Co_2_MnSiC-based glass-coated microwires with an aspect ratio near unity makes them excellent candidates for advanced sensing applications. Additional investigations of Co_2_MnSiC microwires with different aspect ratios and the influence of annealing on the magneto-structural properties of novel Co_2_MnSiC-based glass-coated microwires can reveal the role of internal stresses on the observed thermal stability of magnetic properties.

## Figures and Tables

**Table 1:** The atomic percentage of Co, Mn, Si, and C elemental composition in Co_2_MnSiC and Co_2_MnSi glass-coated microwires.

EDX Spectrum	Av. Co (at.%)	Av. Mn (at.%)	Av. Si (at.%)	Av. C (at.%)
Co_2_MnSi-MWs	51 ± 0.6	23.9 ± 0.5	25.1 ± 0.7	-
Co_2_MnSiC-MWs	50.4 ± 0.2	23.8 ± 0.3	25.4 ± 0.6	0.4 ± 0.1

**Table 2:** The average grain size, lattice parameter, and microstructure order of Co_2_MnSi and Co_2_MnSiC glass-coated microwires.

Parameters	Co_2_MnSi-MWs	Co_2_MnSiC-MWs
D_g_ (nm)	46 ± 0.7	29.2 ± 0.6
a (Å)	5.62	2.85
Order	L2_1_ and B2	A2

**Table 3:** The coercivity and normalized remanent variation with temperature for Co_2_MnSi and Co_2_MnSiC glass-coated microwires.

	Co_2_MnSi-MWs		Co_2_MnSiC-MWs	
T (K)	Hc (Oe)	Mr	Hc (Oe)	Mr
5	7 ± 1	0.22 ± 0.01	19.8 ± 0.5	0.096 ± 0.001
10	6 ±1	0.19 ± 0.01	19.8 ± 0.5	0.1 ± 0.001
20	5 ± 1	0.18 ± 0.01	19.9 ± 0.5	0.096 ± 0.001
50	7 ± 1	0.2 ± 0.01	20 ± 0.5	0.092 ± 0.001
100	6 ± 1	0.2 ± 0.01	20 ± 0.5	0.09 ± 0.001
150	6 ± 1	0.2 ± 0.01	19.9 ± 0.5	0.08 ± 0.001
200	8 ± 1	0.2 ± 0.01	19.8 ± 0.5	0.08 ± 0.001
250	8 ± 1	0.22 ± 0.01	19.8 ± 0.5	0.07 ± 0.001
300	9 ± 1	0.23 ± 0.01	19.6 ± 0.5	0.07 ± 0.001
Δ	4 (Oe)	0.05	0.4 (Oe)	0.03

**Table 4:** The estimation of thermal magnetization stability of FC and FH curves of as-prepared Co_2_MnSiC glass-coated microwire.

H (Oe)	ΔM (%) (FC)	ΔM (%) (FH)	ΔM (%) Av.
50	81	82	81.5
200	91	91.6	91.3
1000	97.3	98.1	97.7
5000	95.2	95.4	95.3
20,000	93.6	93.8	93.7
Av.	91.6	92.2	91.9

## Data Availability

Not applicable.
